# A comparative study of an Yb-doped fiber gain-managed nonlinear amplifier seeded by femtosecond fiber lasers

**DOI:** 10.1038/s41598-021-04420-3

**Published:** 2022-01-10

**Authors:** Dorota Tomaszewska-Rolla, Robert Lindberg, Valdas Pasiskevicius, Fredrik Laurell, Grzegorz Soboń

**Affiliations:** 1 Laser and Fiber Electronics Group, Faculty of Electronics, Photonics and Microsystems, Wrocław University of Science and Technology, Wybrzeże Wyspiańskiego 27, 50-370 Wrocław, Poland; 2 Department of Applied Physics, Royal Institute of Technology, Hannes Alfvéns Väg 11, 106 91, Stockholm, Sweden

**Keywords:** Fibre lasers, Ultrafast lasers

## Abstract

In this work, we show that the nonlinear evolution of femtosecond seed pulses with different parameters (temporal and spectral shapes, repetition rate, pulse energy) in an Yb-fiber amplifier leads to gain-managed nonlinear amplification, enabling robust generation of high-peak-power and nearly transform-limited pulses after external compression. We demonstrate a compressed pulse duration of 33 fs with an energy of 80.5 nJ and a peak power of 2.29 MW for a source with a repetition rate of 30 MHz. For a second seed source with a repetition rate of 125 MHz, we obtained a pulse duration of 51 fs with an energy of 22.8 nJ and a peak power of 420 kW. Numerical simulations incorporating rate equations and nonlinear propagation in the amplifier provide evolutions that agree well with the experimental results. The discrepancies in the amplifier’s absorption edge appearing at low repetition rates and higher pump powers are attributed to the temperature dependence of the amplifier’s gain cross-sections. Here, we experimentally verify this attribution and thus underline the importance of accounting for the fiber core temperature for precise modelling of the short-wavelength spectral edge of the output pulses in nonlinear Yb-fiber amplifiers. We also measure, for the first time, the relative intensity noise of an amplifier operating in the gain-managed nonlinear regime. The measurements reveal a significant contribution of the amplification process to the overall output noise of the system.

## Introduction

Over the past decades, there has been an increased interest in fiber lasers, not only in terms of high-power continuous-wave sources but also for monolithic and robust ultrafast devices, which are attractive for various scientific and industrial applications^1,2^. Furthermore, stabilization of such pulses also opens up for efficient nonlinear conversion to the molecular fingerprint region, which has become a spectroscopic hot-topic in recent years^3,4^. However, the power levels from fiber oscillators are typically inadequate to directly meet the necessary requirements, and subsequent amplification is thus required. The most common approach is chirped-pulse amplification (CPA)^5^, where the pulse is first stretched in time, then amplified, and lastly recompressed. Although the layouts of CPA systems are pretty simple and offer favourable power scalability, they conventionally require negligible nonlinear effects, which puts limitations on the achievable pulse energies in standard fibers with small mode areas^6^. Furthermore, dispersion mismatches between fiber stretchers and grating compressors limit the compressibility of the pulses after amplification^7^. This can, however, be compensated if a sufficiently large nonlinear phase is accumulated during the amplification^7^, but this requires a quite precise balancing. Additionally, gain narrowing can also limit the achievable bandwidths and hence compressed pulse durations^7^. However, these systems can be complemented with a nonlinear pulse compression stage^8^ to reach a shorter pulse duration at the expense of increasing the system’s complexity.

There are, however, techniques that exploit nonlinear effects during the amplification process such that the amplified pulses can be directly compressed below 100 fs. The two main approaches rely on parabolic pulses^9,10^ and pre-chirp management^11,12^. Nonetheless, these do require balancing several parameters and can also need additional steps^12^. In contrast to these approaches, the recently discovered gain-managed nonlinear (GMN) amplification regime^13^ relies on a nonlinear attractor, making it possible to use a great variety of input pulses. This results from the interplay between self-phase modulation and a longitudinally varying asymmetric gain profile that gradually redshifts the spectrum during the amplification process under normal dispersion. To date, state-of-the-art GMN systems have achieved 38 fs pulses with energies of 1.2 μJ^14^.

Regardless of the amplification scheme, a universal strategy for increasing the pulse energy is to reduce the repetition rate of the seed source. However, this approach poses a greater risk of accumulating amplified spontaneous emission in between consecutive pulses, as well as contributes to the thermal load on the fiber upon reabsorption. Furthermore, higher pulse energies can lead to increased peak power levels and consequently stronger spectral broadening. As the pulse gradually redshifts in the GMN regime, its evolution is associated with short-wavelength absorption, which could also increase the fiber’s thermal load and potentially influence the pulse evolution. Therefore, in this work, we investigate the impact of seeding a GMN amplifier with two different lasers, operating at repetition rates of 125 MHz and 30 MHz. We observed significantly better performance in terms of pulse compressibility for the lower repetition rate, which is attributed to a stronger nonlinear spectral broadening during the amplification. We found that at low repetition rates and high pumping power, the gain profile of the Yb-doped fiber amplifier shifts towards longer wavelengths, favourably influencing the GMN amplification. The shortest obtained pulse after amplification at 30 MHz repetition rate had a duration of 33 fs which is, to our knowledge, the shortest pulse duration reported to date for a GMN amplifier.

## Results and discussion

**Figure 1:**
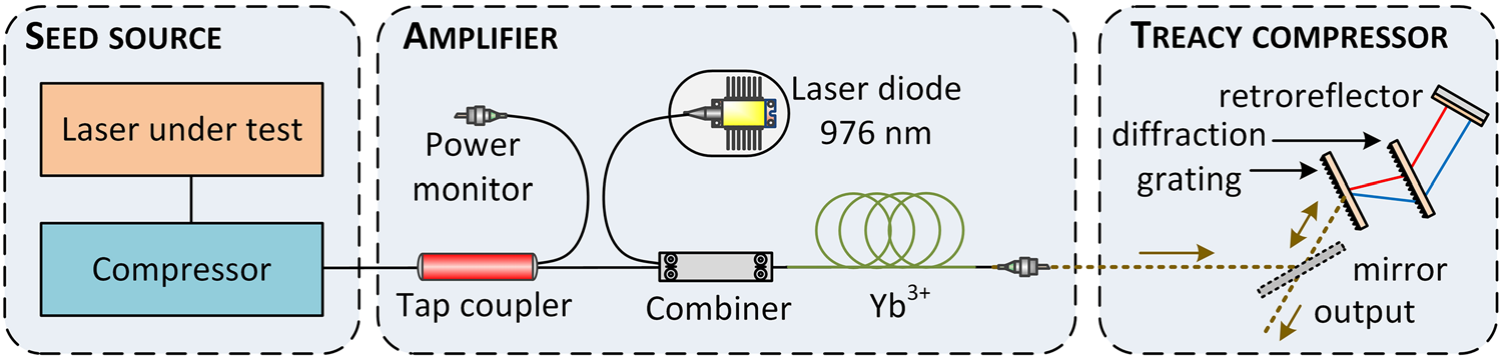
Schematic of the gain-managed nonlinear amplification system.

The experimental setup of the ultrashort pulse amplifier is depicted in Fig. 1. It consisted of polarization-maintaining (PM) fibers and components: a 90/10% tap coupler for power monitoring (Advanced Fiber Resources), a pump-signal combiner (Advanced Fiber Resources), and a 3.4-m-long double-clad Ytterbium-doped active fiber with a 10-μm core diameter (Nufern PLMA-YDF-10/125-VIII) pumped by a 976 nm laser diode (K976A02RN-9.000 W, BWT). The final compressor was of Treacy type^15^ and was implemented with two transmission gratings (1000 lines/mm) that gave an overall transmission of 75%.

**Figure 2:**
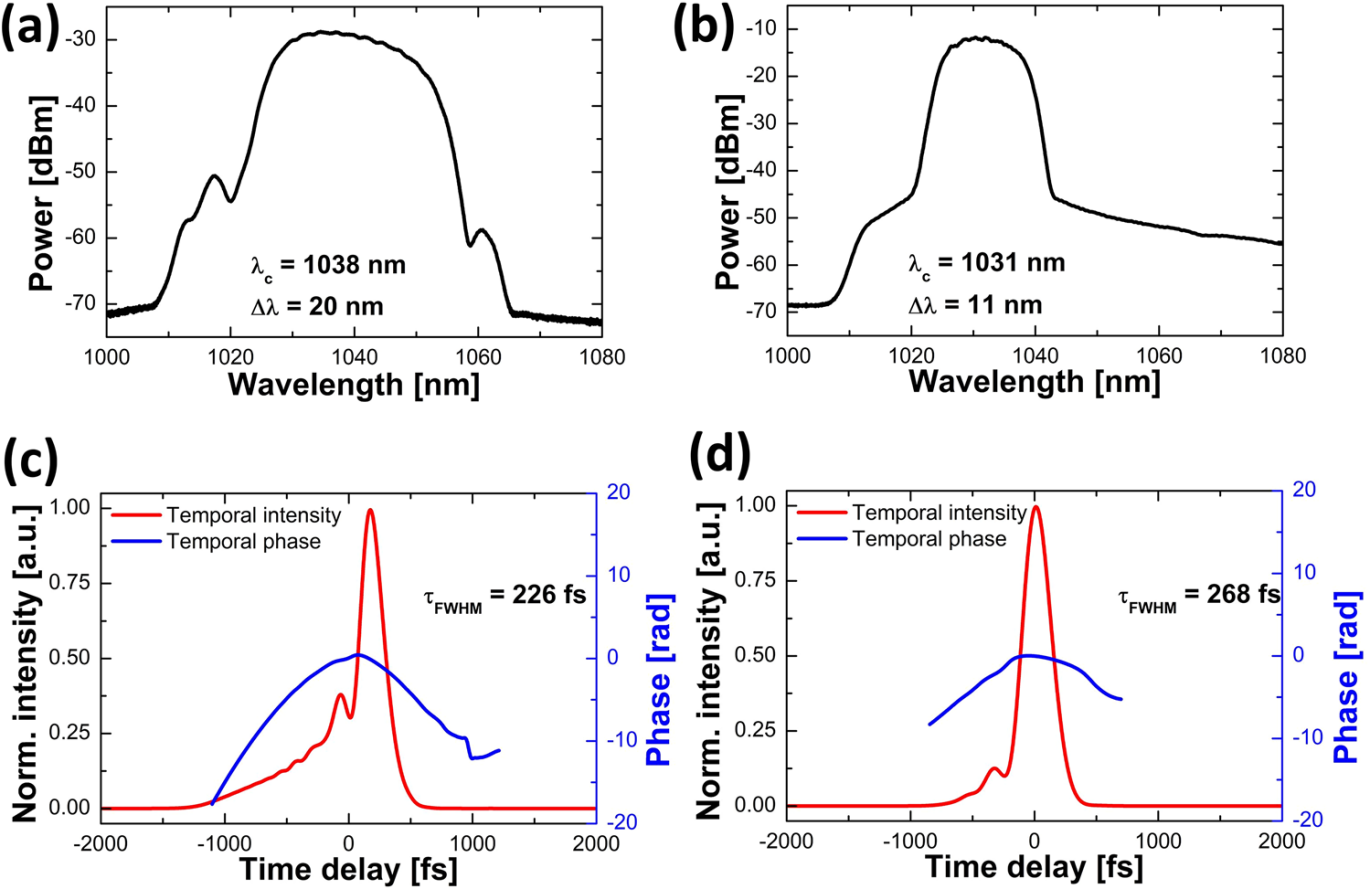
Characterization of seed pulses launched into the amplifier: optical spectrum for (**a**) the high-repetition-rate and (**b**) the low-repetition-rate seed source, together with FROG-retrieved temporal intensity (solid red line) and temporal phase (solid blue line) for (**c**) the high-repetition-rate and (**d**) the low-repetition-rate seed source.

The first explored seed was an optical frequency comb source (Orange, Menlo Systems) based on an Yb-doped fiber laser. The laser emitted chirped 10 ps-long pulses at a central wavelength of 1038 nm, with 20 nm optical bandwidth, 140 mW of average power and a high repetition rate of 125 MHz. The second investigated seed laser was based on a home-built Yb-doped fiber oscillator, mode-locked with a semiconductor saturable absorber mirror (similar to the lasers in previous demonstrations^16,17^), followed by an Yb-doped fiber pre-amplifier which boosts the power to approx 125 mW. This laser emitted chirped pulses with 12 ps duration, centered at 1031 nm with an optical bandwidth of 11 nm, and a low repetition rate of 30.44 MHz. Both the seed sources were compressed before amplification, and the compressor dispersion was adjusted to obtain the shortest pulse duration after the second compressor at the highest pump power—the further presented results all had the same input pulses as shown in Fig. 2. Note that these traces were recorded after the first compressor, and the input pulse to the amplifier is afterwards influenced by the propagation through the pump-signal combiner. These pulses had an average power of 55 mW for high-repetition-rate source and 40 mW for low-repetition-rate source.

### Amplification of the high-repetition-rate laser source

**Figure 3:**
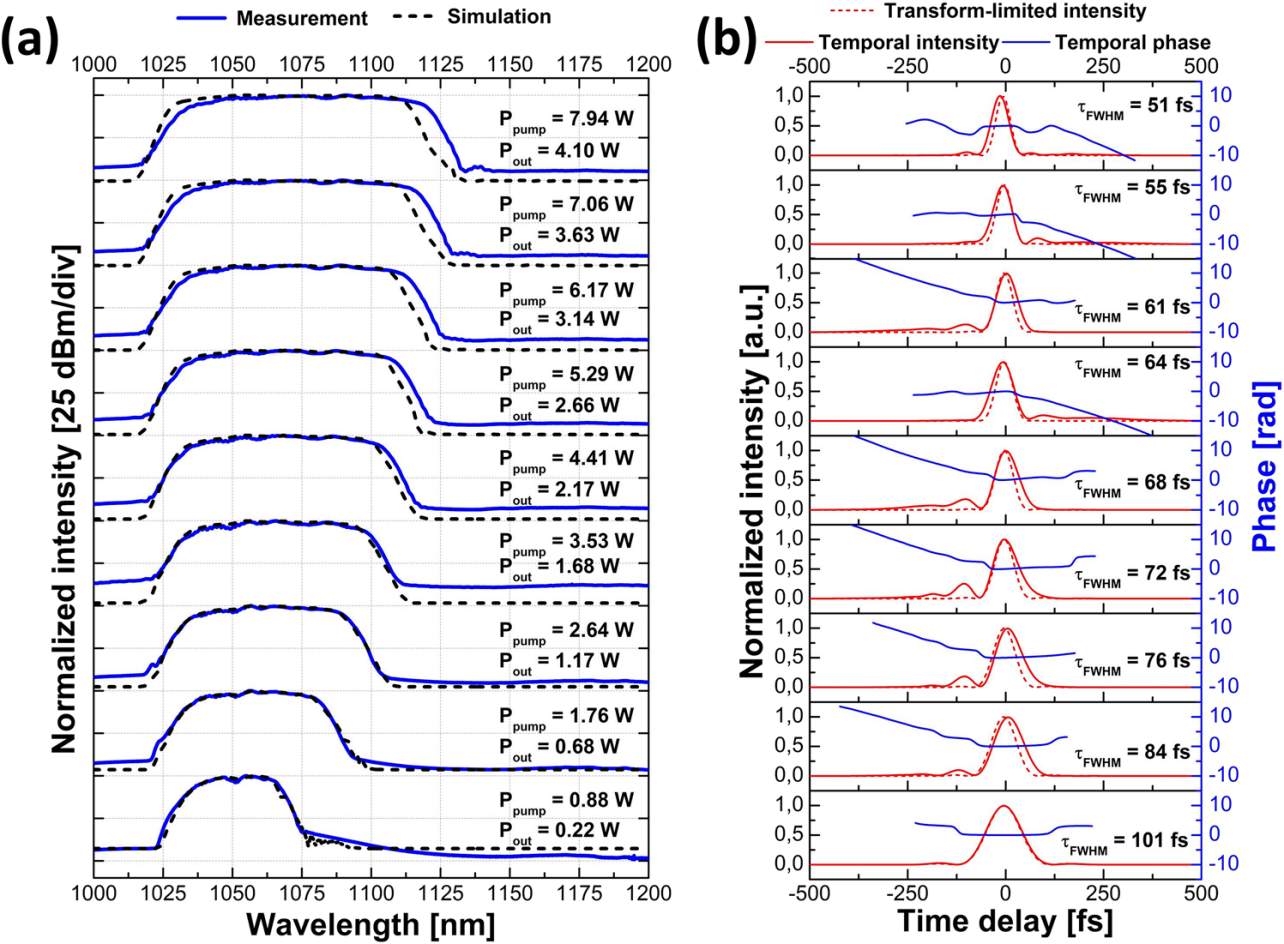
Characterization of the amplified pulses for the high-repetition-rate laser source: (**a**) comparison of the simulated spectra at the output of the amplifier (dashed black line) and traces measured with the OSA (solid blue line) with the corresponding pump powers and average output powers after the amplifier; (**b**) FROG-retrieved temporal intensity (solid red line) of the compressed pulses together with temporal phase (solid blue line), transform-limited intensity (dashed red line) and the corresponding pulse duration.

A comparison between the simulated and the recorded spectra for different pump powers at the amplifier output (before the final compressor) is shown in Fig. 3a for the high-repetition-rate source together with the pump power and the average output power. The agreement between the experimental and the simulated spectra is very good, although it is seen that the measured spectra are shifted slightly more towards longer wavelengths at higher power levels than the corresponding simulated traces. The spectral widths do, however, remain comparable. After amplification, the pulses were compressed in a diffraction grating compressor to confirm the possibility of generating clean, nearly transform-limited pulses from the GMN amplifier. For each pump power, the grating separation was fine-tuned to achieve the shortest pulse duration. Temporal intensities measured via FROG together with transform-limited, calculated from the magnitude of the FROG-retrieved spectra, intensities, and temporal phase are depicted in Fig. 3b for different pump powers. It is clear that the pulse duration decreases with increasing pump power. It is also seen that the phases over the main part of the pulse are relatively flat, which shows that the compression resulted in nearly transform-limited pulses.

**Figure 4:**
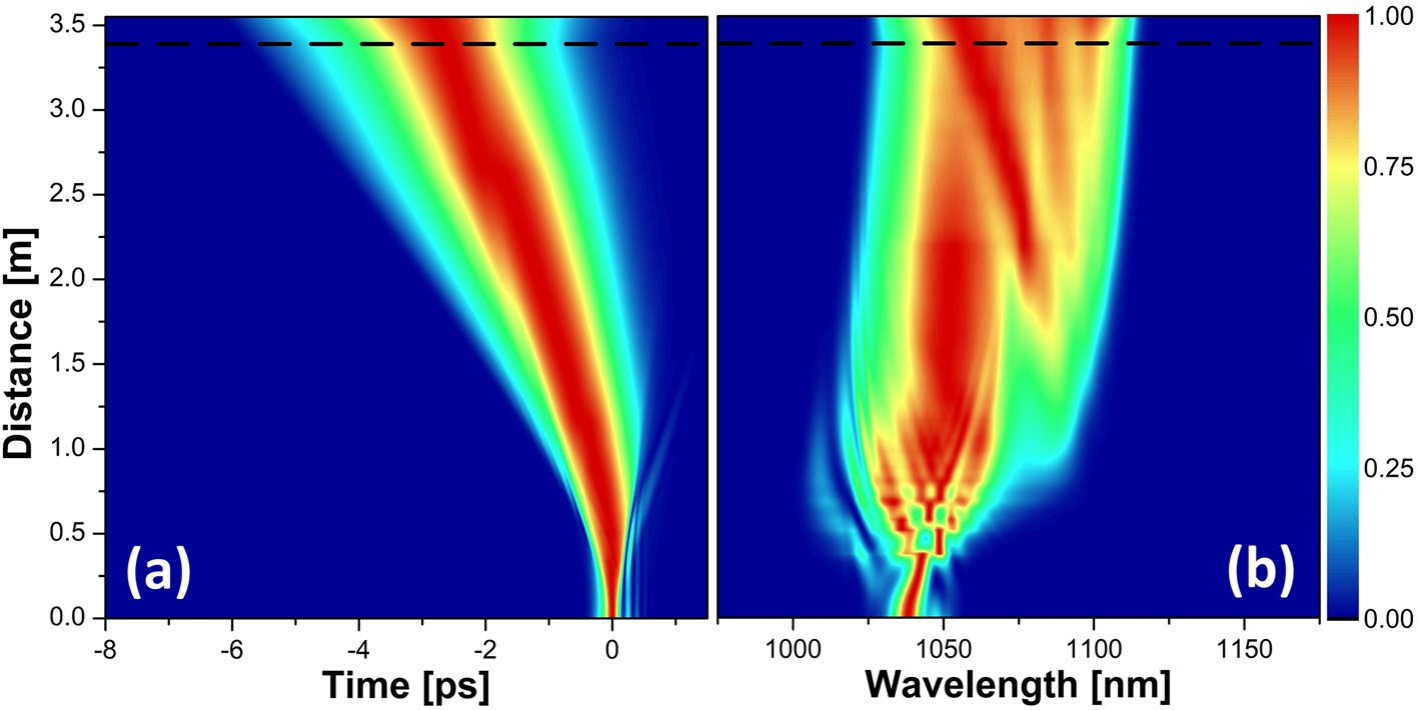
Evolutions of the normalized temporal (**a**) and spectral (**b**) intensities at the highest pump power for the high-repetition-rate laser as a seed source. The dashed black line shows the splice between the active and the passive fiber.

The temporal and spectral evolutions of the pulse in the amplifier and the passive fiber at the highest pump power are shown in Fig. 4. It is seen that the initial part up until about 30 cm is mainly characterized by a spectral shift before the onset of spectral broadening. This stems from the input pulse chirp, which initially cancels the chirp induced from self-phase modulation (SPM). After that, the spectrum broadens quite symmetrically until just below 75 cm, at which point it starts a gradual shift towards longer wavelengths while continuously broadening due to SPM. This shift in the spectral center of mass results from the reduced inversion levels down the fiber, which moves the gain towards longer wavelengths. Consequently, the short-wavelength features forming at the beginning of the fiber are gradually absorbed until about 1.1 m. In the temporal evolution, these spectral parts are indicated by the weak blue arm towards positive time values that fades away into the background at the same distance. It is evident that the overall temporal evolution is characterized by a broadening curved trajectory, which is associated with a spectral broadening in the presence of dispersion in conjunction with a shifting spectral center of mass—since the temporal reference frame is set with respect to the input wavelength. It may also be noted that the temporal evolution becomes rather monotonic after about 1.2 m, which marks the position where the input pulse has been reshaped into a GMN-pulse. The pulse evolutions at lower powers follow the same trend with a spectral broadening accompanied by a shift in the center of mass (which is indicative of the GMN regime^13,14^). Still, the onset of spectral broadening happens at further distances away from the input end, and the overall spectra are hence narrower.

**Figure 5:**
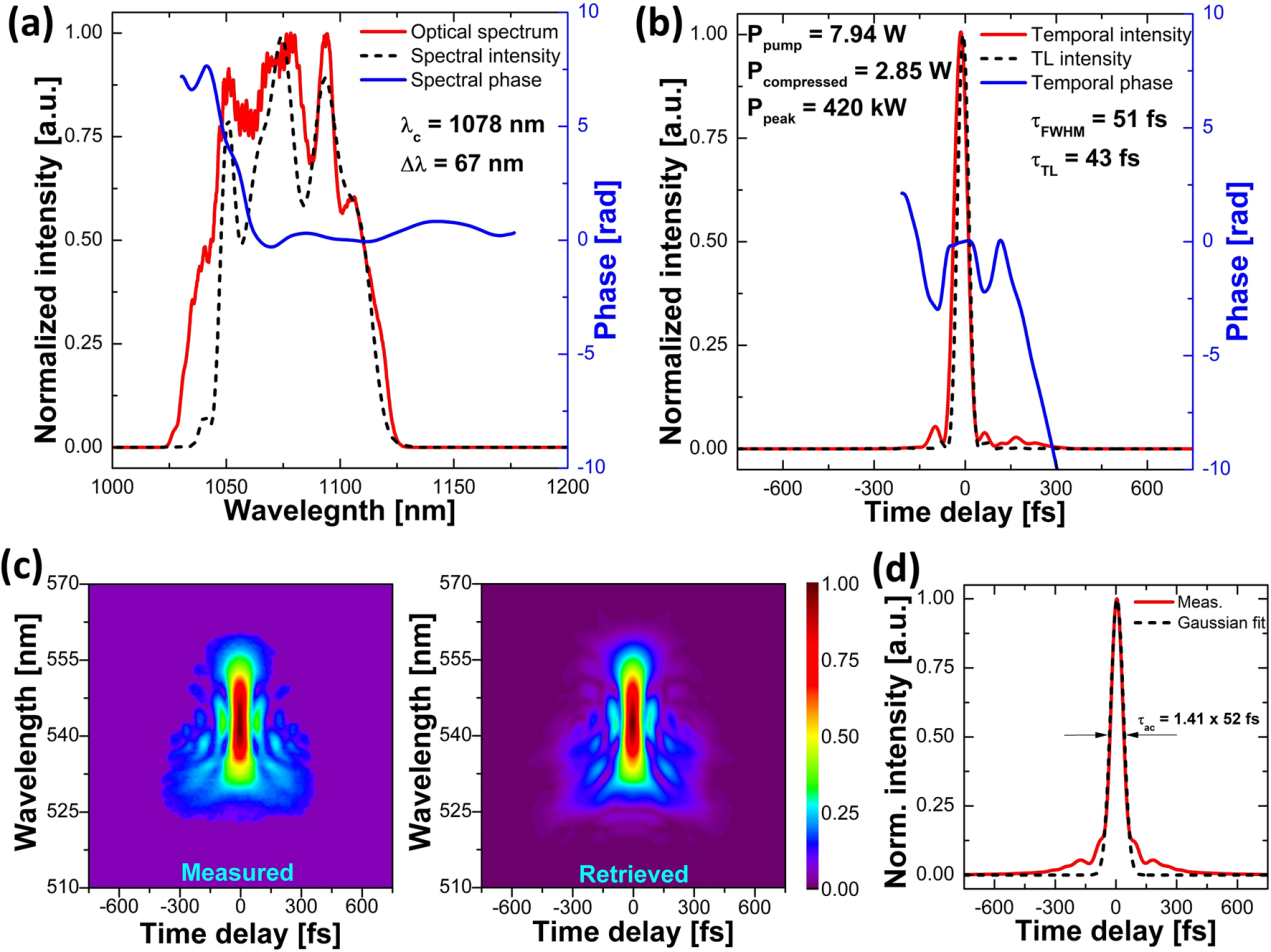
Characterization of the shortest compressed pulse obtained from the GMN amplifier seeded with the high-repetition-rate laser source: (**a**) optical spectrum measured by the OSA (solid red line) and the FROG-retrieved spectral profile (dashed black line) with spectral phase (solid blue line), (**b**) FROG-retrieved temporal intensity (solid red line) of the compressed pulse and temporal phase (solid blue line) as well as transform-limited intensity profile (dashed black line), (**c**) measured and retrieved FROG spectrograms, (**d**) autocorrelation trace (solid red line) and Gaussian fit (dashed black line).

The shortest pulse was obtained at the highest pump power of 7.94 W and was equal to 51 fs (FROG-retrieved measurement), with a calculated transform-limit duration of 43 fs and an average power of 2.85 W measured after the compressor. This corresponds to a calculated pulse energy of 22.8 nJ and a peak power of 420 kW. The central wavelength of this pulse was 1078 nm, and it had a bandwidth of 67 nm. All of the recorded characteristics for this pulse are presented in Fig. 5. It consists of the optical spectrum measured by the OSA, alongside FROG-retrieved spectral and temporal pulse and phase profiles, measured and retrieved FROG-traces, and an autocorrelation trace.

### Amplification of the low-repetition-rate laser source

**Figure 6:**
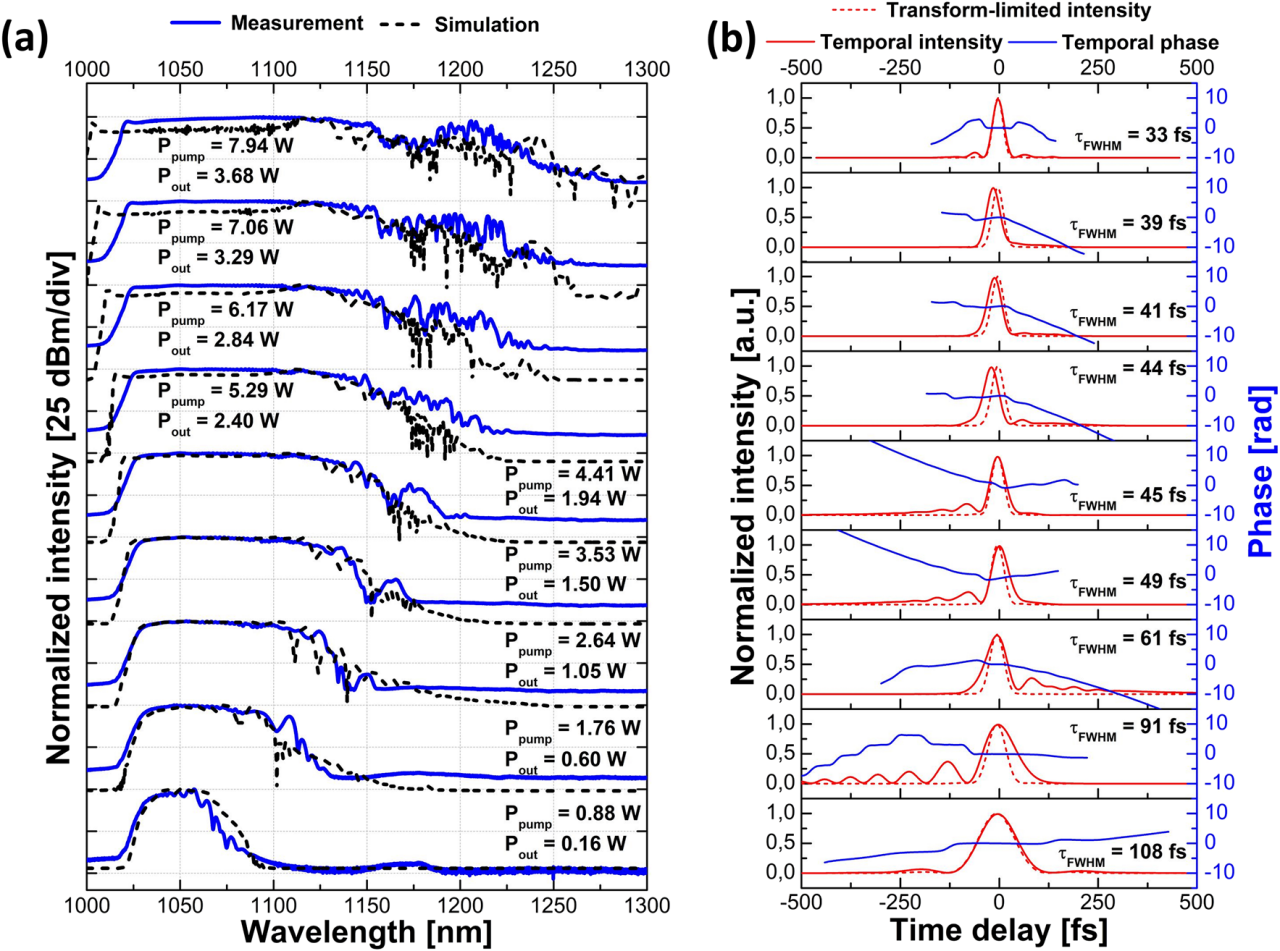
Characterization of the amplified pulses for the low-repetition-rate laser source: (**a**) comparison of the simulated spectra at the output of the amplifier (dashed black line) and traces measured with the OSA (solid blue line) with the corresponding pump powers and average output powers after the amplifier; (**b**) FROG-retrieved temporal intensity (solid red line) of the compressed pulses together with temporal phase (solid blue line), transform-limited intensity (dashed red line) and the corresponding pulse duration.

The evolution of the amplified spectrum as a function of the pump power for the low-repetition-rate seed source after the amplifier is shown in Fig. 6a alongside pump powers and average powers at the output of the amplifier. Temporal intensities of the compressed pulses together with calculated transform-limited intensities and temporal phases are depicted in Fig. 6b. The FROG-retrieved phases are relatively flat over the main portion of the pulse, showing again that they were close to being transform-limited. The obtained spectra for this seed source are broader than those for the high-repetition-rate source and therefore support shorter pulse durations. The rapid oscillations on the long-wavelength edge of the output spectra result from interference between secondary pulse structures separated in time around this wavelength region that appears due to Raman scattering. From Fig. 6a, it is clear that the simulated and the measured spectral shapes start deviating significantly at higher output powers. However, it should be noted that the greatest pulse energy for the high-repetition-rate source was 33 nJ, which is already exceeded at a pump power of 2.64 W with the low-repetition-rate source—and the simulations at this point are still in good agreement with the experimental results. Above pump powers of 4.41 W, the measured shorter wavelength edge is more shifted towards longer wavelengths than the simulated counterparts. As the input pulses remain the same at all applied pump power levels, this cannot be related to an experimentally shorter seed pulse that would start shifting at an earlier point in the fiber—since this would give rise deviations at all power levels. Given that the pulse propagates in the normal dispersion regime, one might suspect transient gain saturation to deplete the gain for the trailing shorter wavelengths. However, both of our sources have MHz-repetition rates and thus far exceed the pump rate of ~ 4.5 kHz (estimated with the recovery time formula in the previous work^18^) such that this effect should only be minor after the passage of many pulses. Furthermore, artificially decreasing the saturation energy by two orders of magnitude did not shift the spectral edge sufficiently to make up for the observed deviation—this also hampered the spectral broadening towards longer wavelengths.

It thus appears that the experimental gain profile gradually starts deviating from the simulated one at higher pump powers, leading to an underestimation of the short-wavelength absorption. This could result from an increased thermal load in the fiber, which was not accounted for in the simulations, due to increased short-wavelength absorption for greater spectral broadening, as well as an increased amount of spontaneously emitted light that gets reabsorbed along the fiber as the pump level increases. Both of these mechanisms effectively act as short-wavelength pumps for emission at longer wavelengths and are thus associated with a reduction in photon energies, with the differing energy being dissipated as heat which leads to an increased thermal load in the fiber.

**Figure 7:**
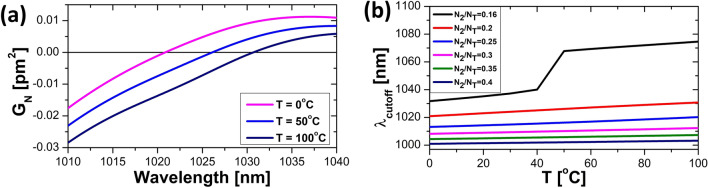
The normalized gain for an inversion level, N_2_/N_T_, of 0.2 at the indicated temperatures (**a**). The shift of the lower wavelength edge of the normalized gain for different inversion levels at various temperatures (**b**).

To illustrate the thermal impact on gain profiles, we plot the normalized gain^19^ in Fig. 7a at different temperatures, using the thermally dependent spectroscopic data for the Yb-doped fiber characterized previously^20^, for an inversion level of N_2_/N_T_ = 0.2. The normalized gain is defined as:

1 $$\begin{array}{*{20}c} {G_{N} = \left( {\sigma_{a} + \sigma_{e} } \right)\frac{{N_{2} }}{{N_{T} }} - \sigma_{a} ,} \\ \end{array}$$

where *σ*_a_ and *σ*_e_ are the absorption and emission cross-sections, *N*_2_ and *N*_T_, respectively denote the upper-level population and the doping concentration. It is clearly seen that the shorter wavelength edge for net gain (i.e., values above 0) shifts towards longer wavelengths at elevated temperatures. The location of the short-wavelength edge of the normalized gain at different temperatures and inversion levels are shown in Fig. 7b, where it is seen that they all shift towards longer wavelengths. However, it should be mentioned that for inversion levels above 0.6, the short-wavelength edge shifts towards shorter wavelengths with increased temperatures. Such high inversions correspond to situations where the main gain is located below 1000 nm and does not apply to the systems studied here.

**Figure 8:**
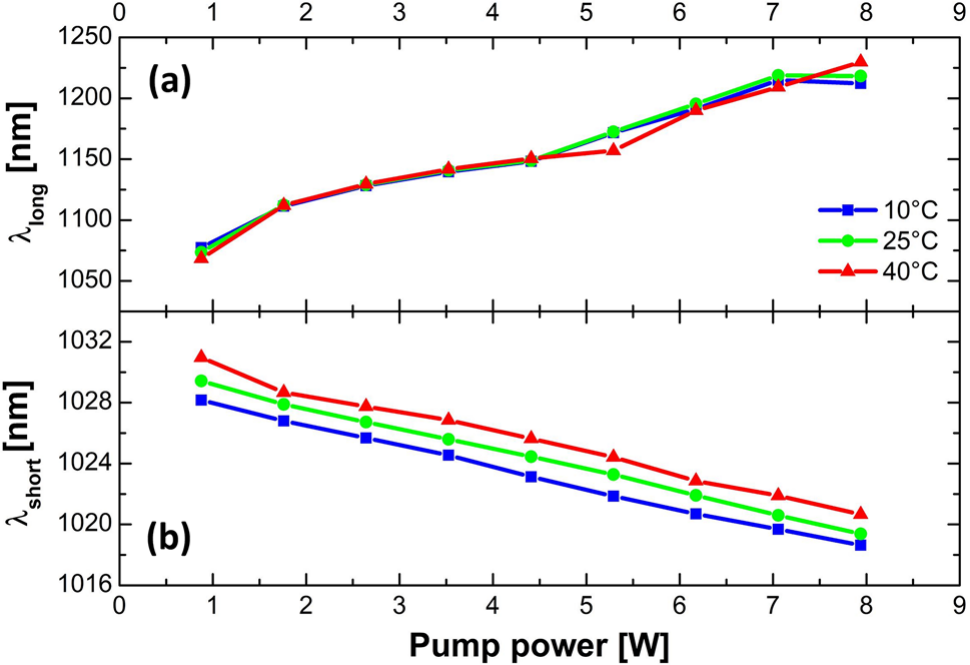
Location of spectral edges in the optical spectrum of amplified pulses in GMN regime, where the intensity equals 1/10 of the maximal intensity for three different temperatures of the cooling plate: (**a**) long-wavelength edge, (**b**) short-wavelength edge.

Unfortunately, we do not have access to all of the necessary fiber parameters to make a quantitative estimate of the amount of heat load to expect. Yet, the temperature distribution in the fiber also depends on how effectively the heat can be dissipated from the fiber. We, therefore, mounted the gain fiber on a water-cooled block and changed its temperature while studying the output spectra at different pump power levels. These results are shown in Fig. 8, where the bottom panel shows how the location of the short-wavelength edge of the spectrum (defined as the point with a spectral intensity of 1/10 of the maximum intensity) varies. It is clearly seen that it shifts to longer wavelengths as the temperature of the cooling block is increased, which also reduces the heat dissipation from the fiber and results in higher core temperatures. The top panel shows that the long-wavelength edge (also defined as the point with a spectral intensity of 1/10 of the maximum spectral intensity) of the spectrum is not affected as much. These findings show that the impact of the thermal load on the gain profile cannot be ruled out as the cause for the discrepancy between the measured and simulated results at the higher pump levels.

The simulated spectra (Fig. 6a) at pump power levels exceeding 4.4 W mostly appear like asymmetric SPM spectra, whereas the experimental ones have similar shapes to the high-repetition-rate source case. In the experiments, they likely undergo a similar spectral evolution with a shifting spectral center of mass that allows the center part of the spectrum to maintain a significant amount of the energy, such as the shapes associated with gain-managed nonlinear amplification^13^. However, it is seen that there appear non-negligible Raman contributions in the experimental spectra, which could potentially degrade the pulse chirp and inhibit clean compression. Still, as evident in Fig. 6b, this was not observed experimentally. Although the simulation results at the highest pump powers do not have a good correspondence to the experimentally obtained spectra, they also have a non-negligible Raman contribution. Furthermore, the simulated pulse evolutions show that the Raman contributions primarily form secondary pulse structures away from the main temporal pulse shape. In such situations, the Raman part will not limit the compression of the main pulse and will instead show up as a low-intensity background. We, therefore, believe that the experimentally observed Raman contributions formed similar secondary structures.

**Figure 9:**
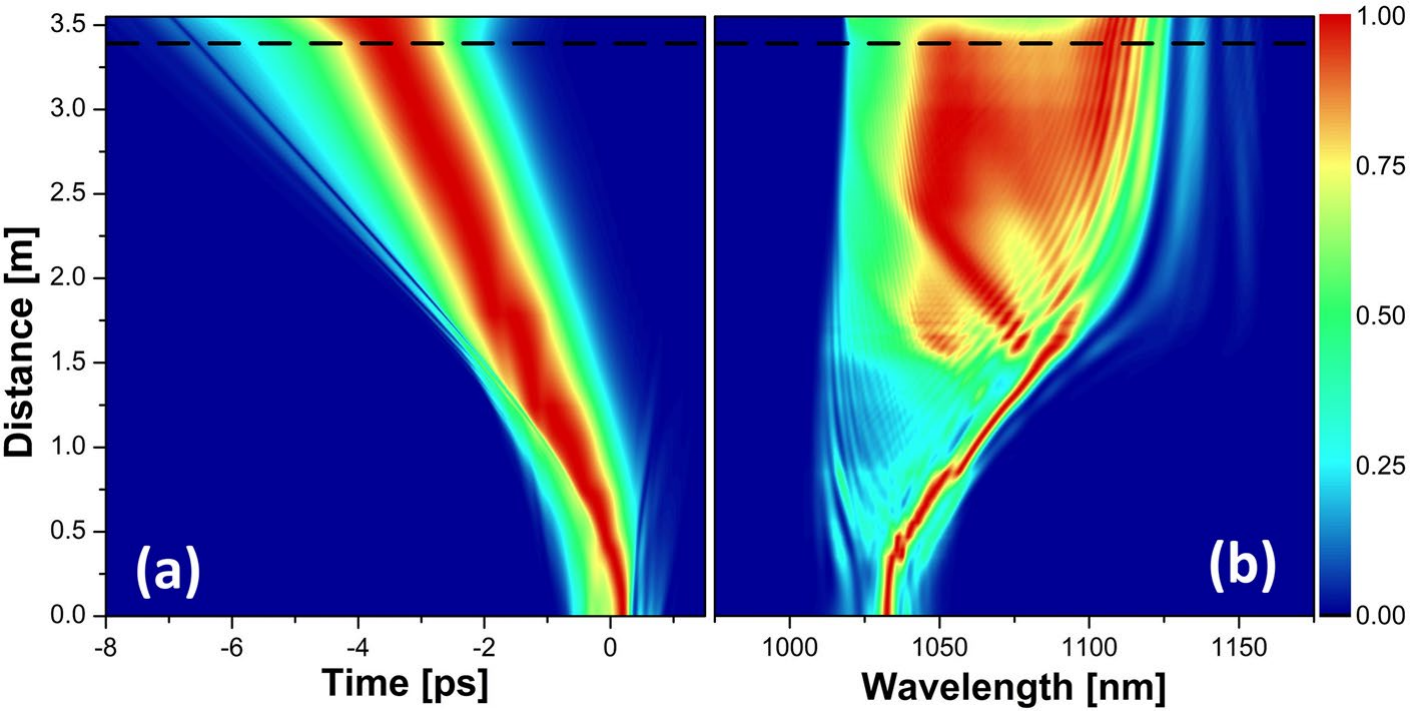
Evolutions of the normalized temporal (**a**) and spectral (**b**) intensities at the 4.4 W of pump power for the low-repetition-rate laser as a seed source. The dashed black line shows the splice between active and passive fiber.

Figure 9 shows the pulse evolution for the low-repetition-rate seed source at a pump level of 4.4 W, where the simulations are still in good agreement with the experimental results. It is seen that just like in Fig. 4, for the high-repetition-rate seed source, the initial evolution is characterized by a temporal reshaping, which in this case is associated with a prominent SPM peak that evolves from the central spectral lobe of the input pulse and gradually shifts towards longer wavelengths. The spectrum settles into a more balanced distribution after about 1.5 m when the gain for the shorter wavelengths starts catching up. Simultaneously, the energy contained in the prominent peak gradually decreases as the Raman contribution grows stronger. It is also at this point that the temporal broadening towards both pulse tails becomes more monotonic, thus marking that the pulse has reached the GMN regime.

**Figure 10:**
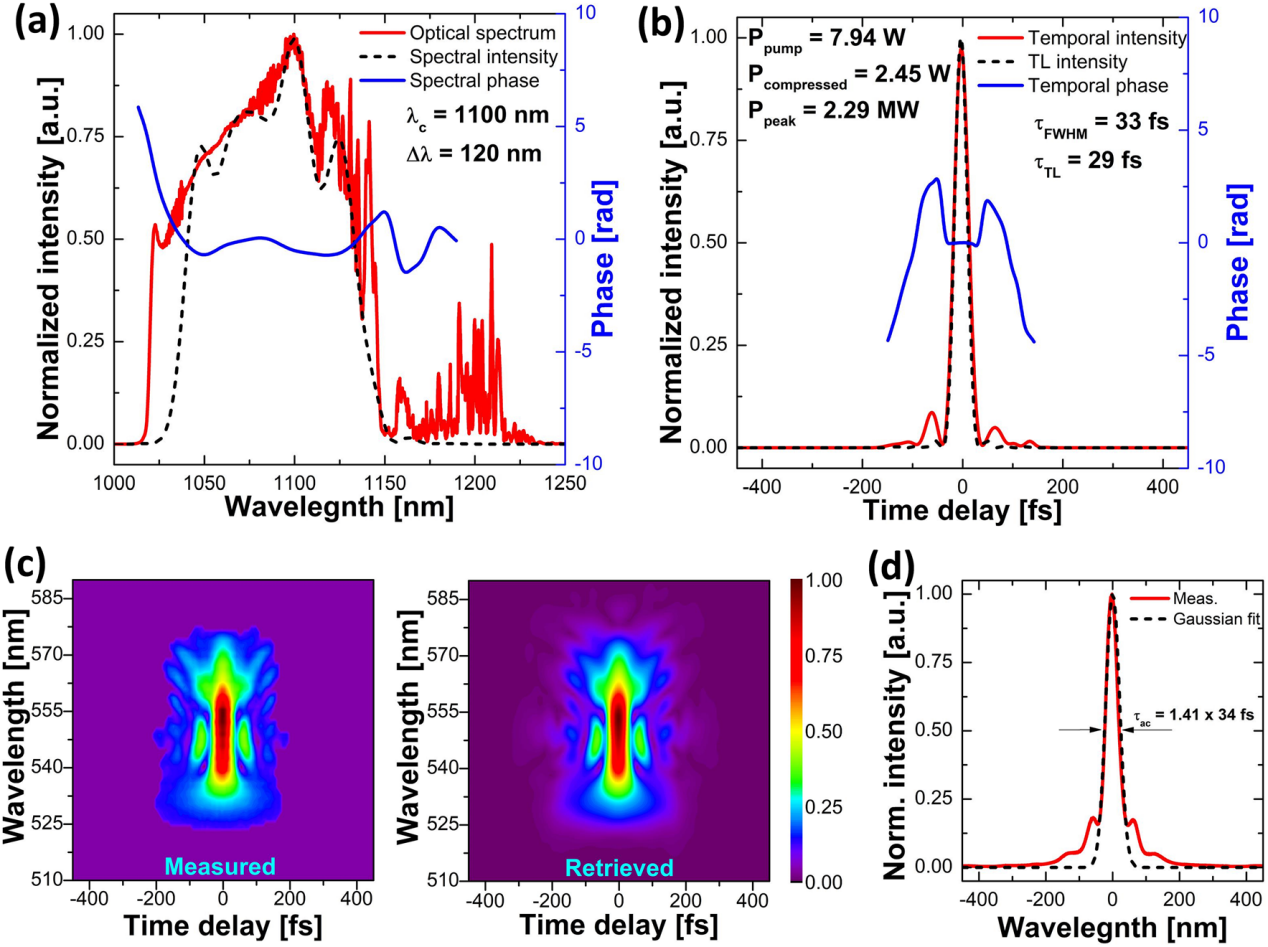
Characterization of the shortest compressed pulse obtained from the GMN amplifier with the low-repetition-rate laser as a seed source: (**a**) optical spectrum measured by the OSA (solid red line) and the FROG-retrieved spectral profile (dashed black line) with spectral phase (solid blue line), (**b**) FROG-retrieved temporal intensity (solid red liane) of the compressed pulse and temporal phase (solid blue line) as well as transform-limited intensity profile (dashed black line), (**c**) measured and retrieved FROG spectrograms, (**d**) autocorrelation trace (solid red line) and Gaussian fit (dashed black line).

The shortest obtained duration for the compressed pulse was 33 fs long, which is only 4 fs from the corresponding transform-limit. This pulse had a power of 2.45 W after the compressor, and its pulse energy equals 80.5 nJ. Peak power reaches 2.29 MW. The central wavelength of the pulse was 1100 nm with a bandwidth of 121 nm. The recorded data for this pulse is shown in Fig. 10, which presents the spectral and temporal pulse and phase profiles, as well as measured and retrieved FROG-traces, an OSA spectrum, and an autocorrelation trace. It should, however, be noted that the FROG-retrieved pulse spectrum is somewhat narrower than the OSA-measured spectrum and does not contain the Raman contribution. As mentioned before, it is likely that the Raman part does not compress properly and appears as a low-intensity dispersive background, which will not be measurable by FROG after the process of second-harmonic generation. The obtained pulse duration of 33 fs is shorter than the ones reported so far^13,14^, making this, to our knowledge, the shortest pulse achieved for GMN amplification to date. The pulse energy of 120 nJ before compression is also higher than the one recorded in the first demonstration of GMN amplification^13^ but lower than reported by Sidorenko et al.^14^, where the authors used a large mode area fiber in the amplifier.

### Relative intensity noise measurements

**Figure 11:**
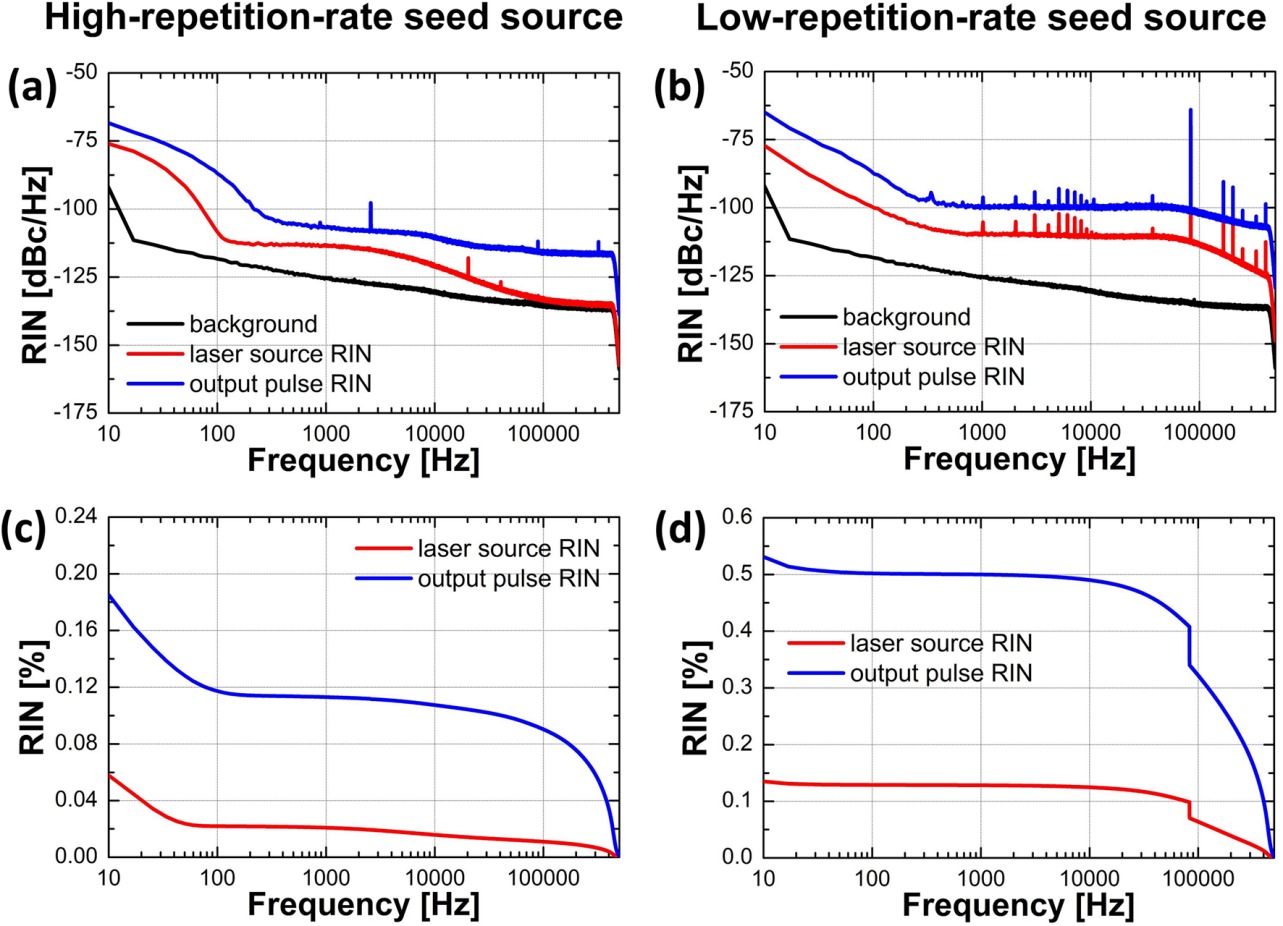
Characterization of the intensity noise of laser sources (red) and also amplified and compressed output pulses (blue): RIN results for (**a**) the high-repetition-rate and (**b**) the low-repetition-rate seed source, together with integrated RIN for (**c**) the high-repetition-rate and (**d**) the low-repetition-rate seed source.

To characterize the noise properties of the GMN amplifier, we performed relative intensity noise (RIN) and integrated RIN rms measurements for both seed sources (high- and low-repetition-rate) at the highest pump power of the amplifier after the final compressor. To determine what is the contribution of the GMN amplification process to the overall noise, we also measured the RIN of the seed sources only. The results are presented in Fig. 11. It can be noticed that the integrated RIN for the high-repetition-rate laser is much lower (0.078%) than for the low-repetition-rate laser (0.145%). This difference then influences the integrated RIN values for output pulses, which equal 0.231% and 0.578%, respectively. We attribute the difference to the quality of driving electronics of the seed lasers (the high-repetition-rate laser is a commercial product intended to use as a metrological frequency comb). For both seed sources, the integrated RIN value of the GMN amplified output pulse is at least 3 times higher than the one measured for the seed source. We can conclude that the GMN amplifier significantly increases the noise of the signal. Moreover, it is not an amplitude stable process. It must be noted that it was not the purpose of this work to construct a low-noise amplifier but to characterize a gain-managed nonlinear regime of amplification for two different laser sources.

## Methods

### Characterization

The performance of the amplifier was characterized using an optical spectrum analyzer (Yokogawa AQ6370B, OSA), a second-harmonic frequency-resolved optical gating system (Mesa Photonics FS-Ultra2, FROG), an interferometric autocorrelator (APE pulseCheck, AC), and an optical power meter (Thorlabs PM400 with a S405C thermal head).

### Numerical simulations

Simulations were run for the segment consisting of the active fiber and the subsequent passive fiber (PM980XP, Nufern). These were initiated by a reference FROG-trace, measured before the active fiber, onto which a background noise level of one photon per frequency channel was added, which acted as a seed for Raman scattering. The splice loss between the two fibers was estimated as negligible, and the mode field radii were calculated as half the core diameter in the active fiber and taken from the datasheet for the passive fiber. The numerical model used for simulating the pulse propagation in the active fiber was based on the one presented previously^18^, where the pump-dopant overlap was used as a free parameter to get a comparable power scaling to the experimental one. This value was set to 0.0025 and somewhat exceeds the ratio of the core/inner cladding areas, 0.0021, which is commonly used as an estimation in double-clad fibers. However, it is known that coiling and twisting conditions can affect the pump-dopant overlap^21^. The nonlinear parameter was also used as a free parameter to match the simulated spectral broadening to the experimental results when using the high-repetition-rate laser as a seed. Lumping the fitting into the nonlinear refractive index, i.e., using the previously mentioned mode-field approximation, results in a value of 4 × 10^−20^ m^2^ W^−1^. Pulse propagation in the passive fiber was simulated using the fourth-order Runge–Kutta in the interaction picture technique^22^ together with an adaptive step-size^23^. For the passive PM980XP-fiber, the intensity-dependent refractive index was set to 2.77 × 10^−20^ m^2^ W^−1^, based on the data from Milam^24^, and the dispersion was based on an experimental measurement by the vendor.

### RIN measurements

The measurements followed a procedure similar to the one presented in the literature^25^. A low-noise photodetector (Thorlabs PDA10D2) and an oscilloscope (Rohde-Schwarz RTA4000) were used to characterize noise properties. Every signal was recorded 500 times to reduce noise in the following procedure. First, the recorded signals were converted by Fourier-transformation to the frequency domain and then normalized by the average value. The obtained results presented power spectral density (PSD) over the frequency range 10 Hz–500 kHz. Furthermore, the oversampling method was used to increase the amplitude resolution, as well as a digital filter during oversampled signal calculations and an anti-aliasing filter at the oscilloscope’s input. In the last step, the integrated RIN rms was calculated by integrating PSD over the 10 Hz–500 kHz frequency range.

## Summary

In conclusion, we have presented a comparative study of a GMN amplifier seeded by two lasers with different repetition rates, one commercially available frequency comb source operating at 125 MHz and a home-built mode-locked laser operating at 30 MHz. The amplified pulses from both sources had spectra that extended beyond the conventional Yb-doped fiber gain window up to 1100 nm. The numerical simulations, which confirmed GMN pulse evolutions, were in excellent agreement for the high-repetition-rate laser and lower pump powers with the low-repetition-rate laser. Compression of the amplified pulses resulted in sub-60-femtosecond pulse durations, close to the transform-limits, for both seed lasers. The shortest pulse had a duration of 33 fs with a pulse energy of 80.5 nJ and a peak power of 2.29 MW. To the best of our knowledge, this is the shortest pulse duration reported to date for a GMN amplifier. Moreover, the compressed pulses obtained from the high-repetition-rate source are highly suitable for seeding an optical parametric oscillator for the generation of a mid-infrared frequency comb.

## Data Availability

Data underlying the results presented in this paper are not publicly available at this time but may be obtained from the authors upon request.

## References

[1] Nishizawa N (2014). Ultrashort pulse fiber lasers and their applications. Jpn. J. Appl. Phys..

[2] Fermann ME, Hartl I (2013). Ultrafast fibre lasers. Nat. Photonics.

[3] Schliesser A, Picqué N, Hänsch TW (2012). Mid-infrared frequency combs. Nat. Photonics.

[4] Vainio M, Halonen L (2016). Mid-infrared optical parametric oscillators and frequency combs for molecular spectroscopy. Phys. Chem. Chem. Phys..

[5] Strickland D, Mourou G (1985). Compression of amplified chirped optical pulses. Opt. Commun..

[6] Limpert J, Roser F, Schreiber T, Tunnermann A (2006). High-power ultrafast fiber laser systems. IEEE J. Sel. Top. Quantum Electron..

[7] Kuznetsova L, Wise FW, Kane S, Squier J (2007). Chirped-pulse amplification near the gain-narrowing limit of Yb-doped fiber using a reflection grism compressor. Appl. Phys. B.

[8] Jocher C, Eidam T, Hädrich S, Limpert J, Tünnermann A (2012). Sub 25 fs pulses from solid-core nonlinear compression stage at 250 W of average power. Opt. Lett. OL.

[9] Fermann ME, Kruglov VI, Thomsen BC, Dudley JM, Harvey JD (2000). Self-similar propagation and amplification of parabolic pulses in optical fibers. Phys. Rev. Lett..

[10] Deng Y, Chien C-Y, Fidric BG, Kafka JD (2009). Generation of sub-50 fs pulses from a high-power Yb-doped fiber amplifier. Opt. Lett. OL.

[11] Chen H-W (2012). Optimization of femtosecond Yb-doped fiber amplifiers for high-quality pulse compression. Opt. Express OE.

[12] Song H (2017). Practical 24-fs, 1-μJ, 1-MHz Yb-fiber laser amplification system. Opt. Express OE.

[13] Sidorenko P, Fu W, Wise F (2019). Nonlinear ultrafast fiber amplifiers beyond the gain-narrowing limit. Optica.

[14] Sidorenko P, Wise F (2020). Generation of 1 µJ and 40 fs pulses from a large mode area gain-managed nonlinear amplifier. Opt. Lett..

[15] Treacy E (1969). Optical pulse compression with diffraction gratings. IEEE J. Quantum Electron..

[16] Jang, G. H., Kim, J. H. & Yoon, T. H. Highly-stable Yb-doped fiber laser mode-locked in a regime of SESAM two-photon absorption. in *CLEO: 2011—Laser Applications to Photonic Applications (2011), paper JWA24* JWA24 (Optical Society of America, 2011). 10.1364/CLEO_AT.2011.JWA24

[17] Ortaç B, Plötner M, Limpert J, Tünnermann A (2007). Self-starting passively mode-locked chirped-pulse fiber laser. Opt. Express OE.

[18] Lindberg R, Zeil P, Malmström M, Laurell F, Pasiskevicius V (2016). Accurate modeling of high-repetition rate ultrashort pulse amplification in optical fibers. Sci. Rep..

[19] Lindberg R (2020). Spectral Control of Functional Fiber Sources.

[20] Peng X, Dong L (2008). Temperature dependence of ytterbium-doped fiber amplifiers. J. Opt. Soc. Am. B JOSAB.

[21] Koška P, Peterka P, Doya V (2016). Numerical modeling of pump absorption in coiled and twisted double-clad fibers. IEEE J. Sel. Top. Quantum Electron..

[22] Hult J (2007). A fourth-order Runge–Kutta in the interaction picture method for simulating supercontinuum generation in optical fibers. J. Lightwave Technol..

[23] Heidt AM (2009). Efficient adaptive step size method for the simulation of supercontinuum generation in optical fibers. J. Lightwave Technol. JLT.

[24] Milam D (1998). Review and assessment of measured values of the nonlinear refractive-index coefficient of fused silica. Appl. Opt. AO.

[25] Mayer AS (2020). Flexible all-PM NALM Yb:fiber laser design for frequency comb applications: Operation regimes and their noise properties. Opt. Express OE.

